# A new species of the leafhopper genus *Calodia* Nielson, 1982 (Hemiptera, Cicadellidae, Coelidiinae) from China, with a key to Chinese species

**DOI:** 10.3897/zookeys.466.8680

**Published:** 2014-12-19

**Authors:** Zhihua Fan, Zizhong Li, Xiangsheng Chen

**Affiliations:** 1 Institute of Entomology, Guizhou University; The Provincial Key Laboratory for Agricultural Pest Management of Mountainous Region; 2 Special Key Laboratory for Developing and Utilizing Insect Resources, Guizhou University, Guiyang, Guizhou Province, 550025 China

**Keywords:** This paper describes and illustrates a new *Calodia* leafhopper species from China (Oriental Region), namely *Calodia dentispina* Fan, Li & Chen, **sp. n.** (Cicadellidae: Coelidiinae: Coelidiini) and provides a distribution map for the new species. A key to the Chinese coelidiine genera and species of *Calodia* is also provided. Leafhopper, morphology, taxonomy, distribution

## Introduction

The Oriental leafhopper genus *Calodia* (Cicadellidae: Coelidiinae: Coelidiini) was erected by Nielson (1982) based on *Calodia multipectinata* as its type species from Malaysia. The genus encompasses 60 known species at present, of which 22 species are found in China (Nielson 1982, 1991, 1996; Li 1989; Zhang 1990, 1994; Li and Wang 1991; Cai and Kuoh 1993; Xu and Kuoh 1999). Recently, we discovered one new *Calodia* species from China, which is described, illustrated and mapped in the present paper. A key to the Chinese coelidiine genera and species of *Calodia* is also provided.

## Material and methods

The morphological terminology adopted herein follows Nielson (1982). Photos of external morphology were obtained by Keyence VHX-1000 system. Illustrations of male genitalia were drawn using an Olympus CX41 stereomicroscope, then enhanced by Adobe Illustrator CS6. All pictures were labelled and figs composition in Adobe Photoshop CS5. The type specimens are deposited in the Institute of Entomology, Guizhou University, Guiyang, China (GUGC).

## Taxonomy

### Key to tribes and genera of Coelidiinae from China

(modified from Zhang 1994)

**Table:** 

1	Ventral side of aedeagus with paraphysis; aedeagus simple, without process, gonopore apical (Thagriini)	***Thagria* Melichar**
–	Ventral side of aedeagus without paraphysis; aedeagus with distinct processes or apically with small teeth, gonopore subapical (Coelidiini)	**2**
2	Aedeagus without distinct process, only dorsal side of apical portion of shaft with many small teeth or spines	***Taharana* Nielson**
–	Aedeagus with distinct processes, occasionally dorsal side of apical portion of shaft with small teeth	**3**
3	Apical or subapical part of aedeagal shaft with one distinct process	***Olidiana* McKamey**
–	Apical or subapical part of aedeagal shaft with two or more distinct processes	***Calodia* Nielson**

#### Genus Calodia

Taxon classificationAnimaliaHemipteraCoelidiinae

Nielson

Calodia Nielson 1982: 140

##### Type species.

*Calodia multipectinata* Nielson, 1982

##### Diagnosis.

This genus can be separated from the other Chinese coelidiine genera mainly by the asymmetrical aedeagus without ventral paraphysis and with two or more apical or subapical processes (see key to genera).

##### Distribution.

Oriental Region.

##### Key to species (male) of the genus

***Calodia***

##### from China

(modified from Zhang 1994)

**Table:** 

1	Pygofer side extended into a long lateral caudodorsal process (Nielson 1982: 174, fig. 560)	**2**
–	Pygofer side not extended into a lateral caudodorsal process, posterior margin with a membranous process (Fig. 7)	**4**
2	Subgenital fig with one apical spine; aedeagal shaft with one row of right lateral spines subapically	**3**
–	Subgenital fig without spine apically; aedeagal shaft with one spine-like process near apex and one row of left lateral setae-like spines slightly distad of midlength	***Calodia webbi* (Nielson)**
3	Pygofer side with a long straight sharply pointed caudodorsal process, dorsal margin with one toothed subapical spine; aedeagus with subapical spines widely spaced	***Calodia warei* Nielson**
–	Pygofer side with a long S-shaped and gradually narrowed caudodorsal process, dorsal margin without subapical spine; aedeagus with subapical spines close together	***Calodia yunnanensis* Zhang**
4	Subgenital fig apex tapered or if not tapered with spines (Nielson 1982: 145 fig. 458; 164 fig. 527)	**5**
–	Subgenital fig apex not tapered and without spine (Fig. 8)	**12**
5	Subgenital fig with subapical spines	**6**
–	Subgenital fig without subapical spine	**9**
6	Aedeagus with two long spines closely appressed to midlength of shaft	**7**
–	Aedeagus with row of short spines on each lateral margin, directed laterally	**8**
7	Forewing with three broad yellowish brown bands and two broad fawn bands transversely	***Calodia patricia* (Jacobi)**
–	Forewing only with two broad, infuscate transverse bands	***Calodia flavinota* Cai & Kuoh**
8	Pygofer caudoventral margin with a small digitate process	***Calodia centata* Zhang**
–	Pygofer caudoventral margin without digitate process	***Calodia bispinosa* Nielson**
9	Aedeagus with one apical spine and one or two subapical spines	**10**
–	Aedeagus with several uniseriate spines on each lateral margin	**11**
10	Aedeagus with one apical spine and one subapical spine	***Calodia obliquasimilaris* Zhang**
–	Aedeagus with one apical spine and two subapical spines	***Calodia obliqua* Nielson**
11	Aedeagal processes long, distinctly separated basally	***Calodia spinifera* Zhang**
–	Aedeagal processes short, close basally	***Calodia setulosa* Zhang**
12	Aedeagus with many processes, without secondary spine (Nielson 1982: 195 fig. 642)	**13**
–	Aedeagus with two processes, with or without secondary spines (Fig. 12)	**16**
13	Aedeagal shaft constricted and narrowed at midlength, flattened at apical half, spines mostly on dorsal surface	**14**
–	Aedeagal shaft narrow throughout, apical half tubular, spines on both lateral margins	**15**
14	Style constricted at midlength and apex, expanded subapically, not bifurcate	***Calodia robusta* Nielson**
–	Style base broad, narrowed distally, subapically bifurcate	***Calodia bifurcata* Xu & Kuoh**
15	Aedeagal shaft with two short left lateral spines and four long right lateral spines; gonopore lateral	***Calodia barnesi* Nielson**
–	Aedeagal shaft with many spines of equal length on each side; gonopore dorsal	***Calodia yayeyamae* (Matsumura)**
16	Aedeagal shaft with two processes without secondary spine (Nielson 1982: 148 fig. 469)	**17**
–	Aedeagus with one or two processes with secondary spines (Fig. 12)	**18**
17	Forewing with a narrow flavous band along costa; both aedeagal processes subapical on shaft	***Calodia ostenta* (Distant)**
–	Forewing without band along costa; both aedeagal processes at midlength of shaft	***Calodia longispina* Li & Wang**
18	Style much longer than connective (Zhang 1994: 126 fig. 124J)	**19**
–	Style slightly shorter than connective (Figs 9, 10)	**20**
19	Both aedeagal processes at apex of shaft	***Calodia apicalis* Li**
–	Both aedeagal processes at midlength of shaft	***Calodia harpagota* Zhang**
20	Aedeagal processes arising on same side of shaft (Fig. 11)	**21**
–	Aedeagal processes arising on different sides of shaft (Zhang 1994: 122 fig. 120L)	**22**
21	Pygofer with internal digitate caudoventral processes; aedeagus with lower process about twice as long as upper process (Figs 11–13)	***Calodia dentispina* sp. n.**
–	Pygofer without internal caudoventral process; aedeagus with processes of near equal length	***Calodia guttivena* (Walker)**
22	Pygofer side with one digitate caudoventral process; both aedeagus processes with secondary lateral spines (Zhang 1994: 122 figs 120F, 120L)	***Calodia lii* Zhang**
–	Pygofer side without caudoventral process; aedeagus with one spine with and one without secondary processes (Nielson 1982: 156 figs 498, 501)	***Calodia fusca* (Melichar)**

#### Calodia dentispina

Taxon classificationAnimaliaHemipteraCicadellidae

Fan, Li & Chen sp. n.

http://zoobank.org/BEC80576-FD0E-4807-90EA-5C2D23507CD5

Figs 1 –13

##### Description.

Length (including wings in repose): ♂ 8.1–8.5 mm, ♀ unkown.

**Figures 1–6.**
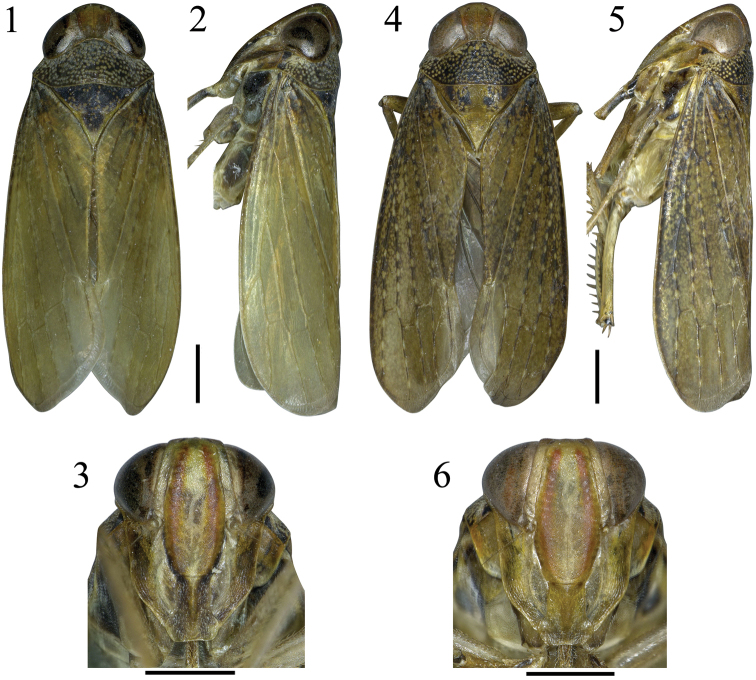
*Calodia dentispina* sp. n. **1–3** Holotype **4–6** Paratype **1, 4** Habitus, dorsal view **2, 5** Habitus, lateral view **3, 6** Facial view. Scale bars = 1 mm.

Crown brown, with a variable red broad band medially, about 1/3^rd^ as wide as midline of crown, ocelli and eyes brown (Figs 1, 4). Face yellow to brown, with a red longitudinal stripe on each lateral margin of clypeus (Figs 3, 6). Pronotum dark brown, with yellow markings (Figs 1, 4). Mesonotum dark brown with yellow spots (Fig. 1) or brown with black spots (Fig. 4). Forewing light brown to brown, with or without yellow patches, venation brown or black (Figs 1, 2, 4, 5).

Head narrower than pronotum; crown longer in middle than next to eyes, length beyond eyes about 1/6^th^ median length, coronal suture extending to level of ocelli, ocelli on anterior margin of crown (Figs 1, 2, 4, 5). Face with clypeus flat, laterally expanded under antennal sockets, apex constricted, clypellus narrow, base inflated longitudinally, apically with lateral margins expanded (Figs 3, 6). Crown, pronotum and mesonotum with ratio along midline about 1:1.3:2 (Fig. 1) or 1:1.1:1.5 (Fig. 4).

**Figures 7–13.**
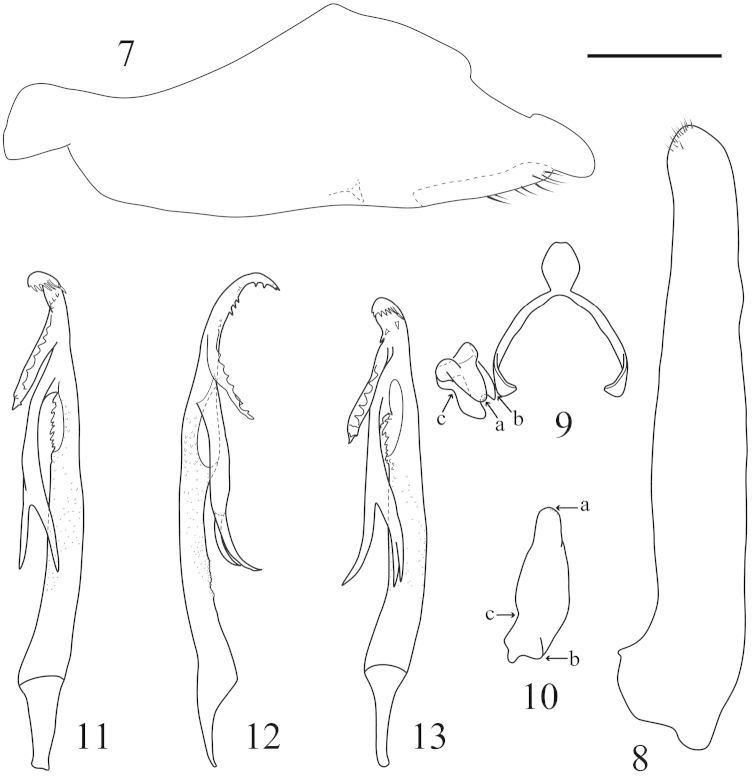
*Calodia dentispina* sp. n., male genitalia. **7–12** Holotype: **7** Pygofer, lateral view **8** Subgential fig, ventral view **9** Connective and style, dorsal view (letters a-c refer to corresponding areas on Fig. 10) **10** Style, dorsolateral view (letters a-c refer to corresponding areas on Fig. 9) **11** Aedeagus, dorsal view **12** Aedeagus, lateral view **13** Paratype: Aedeagus, dorsal view. Scale bar = 0.5 mm.

**Male genitalia.** Pygofer with caudal lobe broadly triangular in lateral view, caudoventral margin inturned with a small internal digitate process (Fig. 7). Segment X without process. Subgenital fig long, apex with short fine setae (Fig. 8). Connective Y-shaped with stem very short (Fig. 9). Style short and simple, apophysis folded at midlength, narrowed distally to rounded apex (Figs 9, 10). Aedeagal shaft asymmetrical, elongate, distally upturned and tapered to acute apex in lateral view with numerous small spines and fine teeth, with two large subapical processes arising on same side, lower process about twice length of other bifurcate apically with inner branch also bifurcate, upper process with margin serrate in lateral view; gonopore large, subapical, situated laterally (Figs 11–13).

##### Distribution.

China (Guangxi).

##### Type material.

Holotype, ♂, CHINA: Guangxi, Chongzuo City, Longzhou County, Nonggang Preserve, 8 May 2012, coll. Fan Zhihua (GUGC). Paratype, 1♂, same data as holotype, except coll. Li Hu (GUGC).

##### Etymology.

The species name *dentispina*, refers to the dentate margin of the shorter aedeagal process.

##### Remarks.

This new species differs from other members of this genus by the shape and configuration of the aedeagal processes.

**Figure 14.**
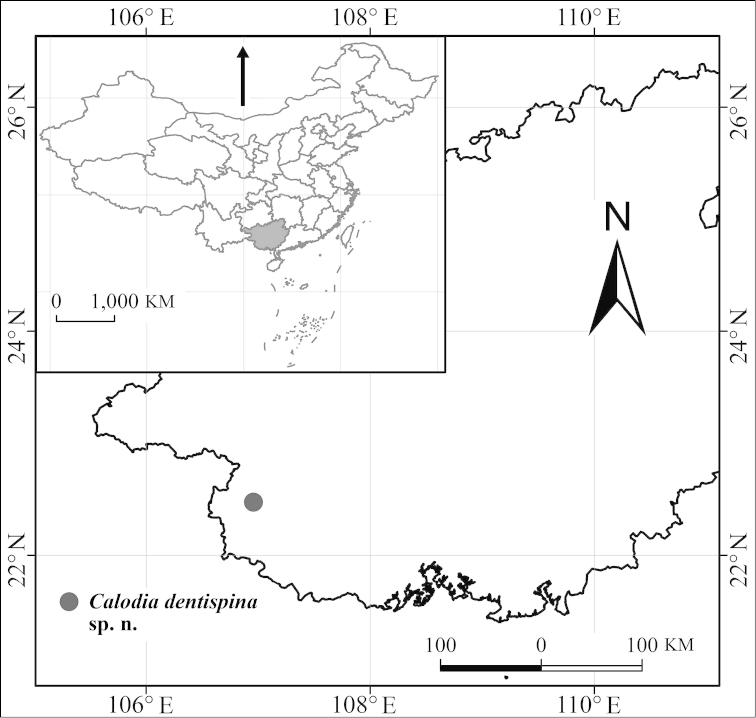
Geographic distribution of *Calodia* new species in China.

## Supplementary Material

XML Treatment for Calodia

XML Treatment for Calodia dentispina
